# Barriers to Enrollment in Rheumatology Research: Who, What, Where, When, and Why?

**DOI:** 10.7759/cureus.27235

**Published:** 2022-07-25

**Authors:** Vaneet K Sandhu, Teodor Duro, Ajay Kamboj, Lorena Salto, Neha Chiruvolu

**Affiliations:** 1 Rheumatology, Loma Linda University Medical Center, Loma Linda, USA; 2 Medicine, Riverside University Health System Medical Center, Moreno Valley, USA; 3 Internal Medicine, Loma Linda University Health, Loma Linda, USA; 4 Care in the Community (CITC) Program, Greater Los Angeles Veterans’ Affairs, Los Angeles, USA; 5 Center for Health Disparities and Molecular Medicine, Loma Linda University School of Medicine, Loma Linda, USA; 6 Internal Medicine, University of California Riverside, Riverside, USA

**Keywords:** population-based research, clinical research work, perceived barriers, rheumatology & autoimmune diseases, barriers to clinical trials enrollment

## Abstract

Despite the evidence that complicated rheumatic diseases are more prevalent and severe in ethnic minorities, rheumatology research is afflicted with inadequate patient representation. It is lacking in ethnic and socioeconomic diversity. The objective of this study is to identify barriers to enrollment in rheumatology research and propose possible solutions to overcome these barriers.

In this study, 184 patients from two rheumatology clinics (Safety Net clinic, a university-based clinic) were surveyed for concerns regarding participation in clinical research. Patients were asked to rank their top five of eight concerns. Data were then stratified by self-reported ethnicity and clinic site to determine ranking differences in both groups.

Fear of risks associated with clinical research was ranked as the primary barrier in all ethnicities. More non-Hispanic Whites (NHW) (24.4%) ranked work responsibilities as a primary barrier compared to Hispanics (10%). Fear of discovering a serious illness as a primary barrier was more frequent at the Safety Net clinic (25%) compared to the university-based clinic (6.3%) and ranked more frequently in the top five in Hispanics compared to NHW.

Fears associated with research risks, work responsibilities, and fear of discovering a serious illness were the top-ranked barriers to enrollment in research among patients. However, differences in rankings between ethnicities and clinic sites were identified. This sheds light on the importance of health literacy and the responsibility of researchers in addressing gaps in communication while acknowledging potential cultural components that warrant further investigation.

## Introduction

Clinical trial research is the cornerstone of advancing novel therapeutics and management strategies in rheumatic disease. The heterogeneity of disease manifestations in complex autoimmune conditions emphasizes the importance of innovative research in rheumatology. Unfortunately, rheumatology research is afflicted with inadequate patient representation. In the United States, clinical research participants are more likely to be non-Hispanic White (NHW) and of higher socioeconomic status, with an inadequate representation of Hispanics, Blacks, Asians, or other ethnic minority groups [1,2]. This missing data is particularly important where the prevalence and prognosis of a disease and its treatment response may vary by ethnicity. For example, the prevalence of systemic lupus erythematosus (SLE) is reportedly 43% in Blacks, 33% in Whites, 16% in Hispanics, and 13% in Asians [1]. Patients from minority communities are more likely to develop severe manifestations of SLE, thereby utilizing greater healthcare resources to control these disease flares [3].

The study of barriers to enrolling ethnic minorities in clinical research has been ongoing for years, and yet it seems much is to be learned from our gaffes. For example, it is not uncommon for researchers to practice culturally insensitive communication and to utilize inappropriate recruitment strategies for different ethnicities [4]. This lack of inclusivity is demonstrated by Tanner and colleagues whose study of clinical trial enrollment strategies concluded that investigators from academic medical centers were not recruiting medically underserved communities [5]. The presence of systemic factors must also be considered, such as access to a higher level of care. For example, most medical centers with specialty providers are located in cities. Patients living in rural areas consequently travel farther to gain access to routine healthcare, thereby reducing the likelihood of research participation by proxy [6]. Similarly, lack of health insurance is more prevalent in minorities and limits access to healthcare and research facilities [7].

Barriers from a participant’s perspective are of equal, if not greater, importance. Thus far, the study of barriers to clinical research enrollment from a patient perspective stems primarily from oncology and infectious disease. One study that surveyed oncology patients on cognitive-affective and practical barriers to clinical research identified fear of side effects as the most common barrier [8]. Another study addressing factors affecting patient participation in clinical trials reported that 49% of patients declined participation; the most common reasons were distance from the cancer center and desire for alternative treatment [9]. The INGENIOUS trial evaluated more than 1,000 patients (>60% minority population) and found that patients frequently cited lack of interest in research, time investment, and distance from the study site as reasons to not enroll [10]. Additional barriers include feeling a loss of control (i.e. study participation = inability to control health), competing demands, mistrust in research, insurance coverage, and more [4,11,12].

The research of such barriers as they pertain to rheumatology clinical research is confined to one study in Romania [13]. Researchers identified patient motivators and barriers to participate in clinical trials with novel therapies for rheumatoid arthritis and identified that the major barrier was the individual’s fear of potential side effects of experimental treatment [13]. This study, however, does not address barriers for the diverse patient populations encountered in the United States. The paucity of studies addressing barriers to clinical research participation in ethnic minority patients with rheumatic disease led to the development of this study.

Our objective was to identify and propose possible solutions to overcome barriers to patient enrollment in rheumatology research. We hypothesized that underserved ethnic groups and patients seen at our Safety Net hospital clinic site would differentially rank barriers to enrollment in rheumatology clinical research compared to NHW and patients seen at our university-based clinic site. This hypothesis largely stems from the greater representation of commercial insurance or self-pay (>70%) in our university-based clinic site compared to >80% public insurance at the Safety Net hospital.

## Technical report

Methodology

This study was approved by the institutional review board at both Loma Linda University Health (LLUH) and Riverside University Health System (RUHS). In total, 184 semi-structured interviews were conducted with patients seen in the rheumatology clinics at LLUH Faculty Medical Clinic (university-based clinic site, FMC) and RUHS clinics, academic private practice-based and community-based models, respectively.

Consecutive patients seen in the rheumatology clinics at LLUH FMC and RUHS from August 2018 to December 2018 were surveyed on self-reported ethnicity and perceived concerns in participating in clinical research. This semi-structured interview comprised provider-guided completion of a questionnaire (Appendix 1) in English or Spanish, whereby the patient was asked to rate, in order of importance, their top five of eight barriers or fears of enrolling in a clinical research study. The eight listed barriers were obtained from a review of the literature on prior patient-perceived barriers to enrolling in clinical research primarily in oncology. While this questionnaire is not validated from prior research, it provides insight as a pilot study into perceived barriers in the San Bernardino and Riverside county region. Rankings were entered and managed electronically. Options for barriers included: (1) Fear of discovering a serious illness; (2) fear of risks associated with clinical research; (3) fear of unneeded treatment or testing; (4) transportation difficulty; (5) work responsibilities; (6) caretaker responsibilities; (7) distrust in the healthcare provider/system; (8) fear of loss of control.

Statistical Analysis

We used SPSS version 26.0 (IBM Corp., Armonk, NY, USA) for statistical analyses. We present descriptive statistics for the overall group but also stratify the data by clinic site (RUHS vs. university-based) and by ethnic group (NHW vs. Hispanics). We summarized the eight categorical barriers for enrollment statements as frequencies and percentages. Because we were interested in determining ranking differences by the clinic and by ethnic group, we transformed each of the barrier statements into three separate ranked binary variables. Each barrier statement had a corresponding Rank 1, Rank 2, and Rank 3 binary variable where 1 = the rank of interest (1, 2, or 3) and 0 = else, including did not rank. We used Pearson’s chi-square to test for independence or Fisher’s exact test and set the type 1 error at α = 0.05 for statistical significance.

Results

Table 1 demonstrates both demographic distribution as well as site enrollment. Among the patients surveyed, 88 (48%) were recruited from RUHS and 96 (52%) from FMC. The majority of patients (65%, n = 119) from both subgroups were Hispanic.

**Table 1:** Patient demographics. *Riverside University Health System, community-based model; **Loma Linda University Health, academic private-practice model.

	Safety Net* (n, %) N = 88	University-based clinic** (n, %) N = 96
White, Hispanic	71 (80.7%)	48 (50.0%)
White, non-Hispanic	7 (8.0%)	34 (35.4%)
Black	6 (6.8%)	12 (12.5%)
Asian/Pacific Islander	4 (4.5%)	2 (2.1%)

Table 2 demonstrates the top three barriers to enrolling in clinical research by site and Table 3 by ethnicity. The number one ranked barrier for not enrolling in clinical research across all ethnicities was the fear of risks associated with clinical research. More RUHS patients (27.3%) than patients from FMC (13.5%) ranked this fear as the second reason for not enrolling in clinical research (p = 0.02).

**Table 2:** Top three Barriers for enrolling in clinical research vs ELSE*: comparisons by clinic site. ^a^Rank of interest (1, 2, or 3) = 1, ELSE = 0 (cases that did not rank the barrier). Only the frequencies for the rank of interest are shown. Percentages may not add to one hundred due to rounding. *p-value < 0.05 and based on the Chi-square test of independence or Fisher’s exact test when cell counts were less than five. UBC = university-based clinic; SNC = Safety Net clinic

	Rank 1			Rank 2			Rank 3		
	UBC, n = 96	SNC, n = 88	P-value	UBC, n = 96	SNC, n = 88	P-value	UBC, n = 96	SNC, n = 88	P-value
Fear of risks associated with clinical research	30 (31%)	24 (27%)	0.554	13 (14%)	24 (27%)	0.020*	14 (15%)	15 (17%)	0.647
Fear of unneeded treatment or testing	15 (16%)	9 (10%)	0.277	26 (27%)	18 (21%)	0.292	12 (13%)	29 (33%)	0.001*
Transportation difficulty	12 (13%)	9 (10%)	0.628	7 (7%)	9 (10%)	0.480	3 (3%)	6 (7%)	0.246
Work responsibilities	18 (19%)	6 (7%)	0.016*	9 (9%)	3 (3%)	0.137	4 (4%)	7 (8%)	0.357
Taking care of others	7 (7%)	9 (10%)	0.480	6 (6%)	7 (8%)	0.652	10 (10%)	5 (6%)	0.241
Distrust	4 (4%)	5 (6%)	0.739	6 (6%)	2 (2%)	0.282	9 (9%)	5 (6%)	0.345
Loss of control	5 (5%)	4 (5%)	0.999	14 (15%)	7 (8%)	0.158	14 (15%)	6 (7%)	0.091
Fear of discovering a serious illness	6 (6%)	22 (25%)	<0.001*	11 (12%)	15 (17%)	0.277	11 (12%)	6 (7%)	0.278

**Table 3:** Top three barriers for enrolling in clinical research vs. ELSE*: comparisons by ethnicity. ^a^Rank of interest (1, 2, or 3) = 1, ELSE = 0 (cases that did not rank the barrier). Only the frequencies for the rank of interest are shown. Percentages may not add to one hundred due to rounding. *p-value < 0.05 and based on the Chi-square test of independence or Fisher’s exact test when cell counts were less than five. NHW = non-Hispanic Whites; His = Hispanics

	Rank 1			Rank 2			Rank 3		
	NHW, n = 41	His, n = 119	P-value	NHW, n = 41	His, n = 119	P-value	NHW, n = 41	His, n = 119	P-value
Fear of risks associated with clinical research	12 (29%)	32 (27%)	0.769	5 (12%)	29 (24%)	0.100	7 (17%)	19 (16%)	0.868
Fear of unneeded treatment or testing	5 (12%)	16 (13%)	0.838	13 (32%)	25 (21%)	0.165	7 (17%)	30 (25%)	0.287
Transportation difficulty	7 (17%)	13 (11%)	0.305	2 (5%)	11 (9%)	0.518	3 (7%)	6 (5%)	0.695
Work responsibilities	10 (24%)	12 (10%)	0.022*	2 (5%)	8 (7%)	0.999	1 (2%)	8 (7%)	0.449
Taking care of others	0 (0%)	13 (11%)	0.040*	2 (5%)	8 (7%)	0.999	5 (12%)	9 (8%)	0.365
Distrust	1 (2%)	6 (5%)	0.679	3 (7%)	4 (3%)	0.374	2 (5%)	10 (8%)	0.732
Loss of control	4 (10%)	3 (3%)	0.072	8 (20%)	12 (10%)	0.115	3 (7%)	16 (13%)	0.406
Fear of discovering a serious illness	3 (7%)	24 (20%)	0.088	4 (10%)	19 (16%)	0.442	3 (7%)	10 (8%)	0.999

Work responsibilities were ranked in the top five barriers to enrolling in clinical research in 43% of university-based clinic patients compared to 41% of RUHS patients. These responsibilities were ranked as the primary barrier in 19% of university-based clinic patients compared to 7% of RUHS patients (p = 0.016).

Fear of discovering a serious illness was ranked as the primary barrier in 25% of patients at RUHS compared to 6.3% at FMC (p < 0.001). More Hispanics overall ranked this barrier in their top five compared to NHW (p = 0.011).

Overall, more NHW (24%) compared to Hispanics (10%) ranked work responsibilities as a primary barrier to enrolling in clinical research (p = 0.022) (Table 2). Thirteen Hispanics (11%) between both sites reported taking care of others as a number one barrier to enrolling in clinical research compared to zero for NHW (p = 0.04 Fisher’s exact test).

## Discussion

Irrespective of ethnicity or site (FMC versus RUHS), we find that the concern for risks related to research is the leading barrier for patients enrolling in clinical research. However, the fear of discovering a serious illness was significantly greater in our Hispanic population compared to other ethnicities as well as patients seen at RUHS compared to FMC. On the other hand, work responsibilities ranked higher in NHW and patients seen in the university-based clinic.

Our findings reiterate the ongoing knowledge gap in patients’ understanding of what clinical research entails and the associated risks. This fear is in some ways expected given the history of medicine and the atrocities committed on humans in the name of research [14]. However, progress in the scientific community has led to the development of institutional review boards and other regulatory committees ensuring the ethical conduct of clinical research.

Researchers and healthcare providers must work to diffuse myths regarding clinical research. The notion that the risks of participating in new research outweigh the benefits of proceeding with currently available treatments must be clarified because this is not always true. In fact, it is not uncommon for research studies to include medications that may not otherwise be available to manage one’s disease. This is particularly applicable in rheumatology, where limited availability of FDA-approved treatments often results in the use of off-label use as justified by clinical research. Finally, it is important to inform patients that clinical research does not always comprise treatment but can be monitoring response to standard therapy, emphasizing the value of their involvement and potential global impact on long-term disease management or outcome evaluation.

Fear is often cited as a psychological factor for not obtaining timely treatment and is a barrier to enrolling in clinical research [15]. In our study, this fear was more prevalent in our Hispanic and underserved population. It is unclear whether this is a cultural phenomenon, a byproduct of socioeconomic class, or a combination of multiple factors. Data collected as part of a cancer opinions survey conducted in Britain showed that emotional barriers, for example, fear of what the clinician may find, were more common in lower socioeconomic status. On the contrary, patients of higher socioeconomic status frequently cited barriers such as time commitment to the study [16]. These findings are like what we see in our population, especially the university-based clinic population that is represented by a higher socioeconomic status in comparison to the Safety Net clinic.

The socioeconomic barrier to health literacy must be recognized. Patients and clinical research participants merit access to credible, easy-to-read, and readily available healthcare resources. Limited English proficiency may create communication gaps between provider and patient, affecting health literacy in our population. Culturally, traditional Hispanics have long valued familism, respect, and religion while the “American Dream” mindset includes awareness of material success, independence, and self-reliance [17]. The fear of discovering a serious illness may stem from this notion that if they are diagnosed with a serious illness, the patient may lose their independence.

We suggest several ways of addressing barriers to clinical research. First, we encourage rheumatologists to personalize their conversations with patients to address their health status directly and why it may be beneficial to enroll in clinical research. This may mean taking time to clarify any questions and fears that the patient may have. We cannot assume every patient has the same understanding of their health to make medical decisions. We must fill this gap in knowledge. Language interpreters should be used whenever required to improve communication and understanding. Multilingual pamphlets are resourceful for recalling information presented by providers. The risks and benefits of clinical research should be clearly stated. Several models have been proposed to improve communication between patients and clinical investigators in regard to enrollment in clinical research. Patient-centered outcomes research highlights the importance of shared decision-making and providing tools to make informed health decisions [18]. Community-Based Participatory Research model where community members and researchers are jointly involved in research to build trust and engagement in the community [18]. These can be incorporated into rheumatology research to help address some of the barriers that patients have ranked highly in this study. Similarly, group seminars have demonstrated improved patient participation in clinical research as they provide an environment for patients to ask questions and clear doubts [19].

The cross-sectional design of this study poses a limitation in establishing cause-and-effect conclusions. The number of Black and Asian respondents was also inadequate to draw conclusions, resulting in an analysis primarily of Hispanics and NHW. We acknowledge that additional barriers to enrolling in clinical research likely exist and might include financial barriers, which were not queried. An additional limitation includes the number of options for barriers and ranking, thereby resulting in a comparison of the top three ranked barriers in many cases. In the same vein, the listed perceived barriers may have missed additional barriers pertinent to this population of patients.

## Conclusions

Clinical research in rheumatology needs better demographic representation. Rheumatic diseases disparately affect persons of color and lower socioeconomic status, emphasizing the importance of enrolling a diverse population into clinical studies such that generalizability to the results and conclusions can truly be availed. This study highlights the importance of understanding cultural and socioeconomic differences to address such barriers to recruitment in rheumatology clinical research in addition to the need to inform patients of the importance of clinical research in representative populations, particularly ethnic minorities.

## Figures and Tables

**Figure 1:**
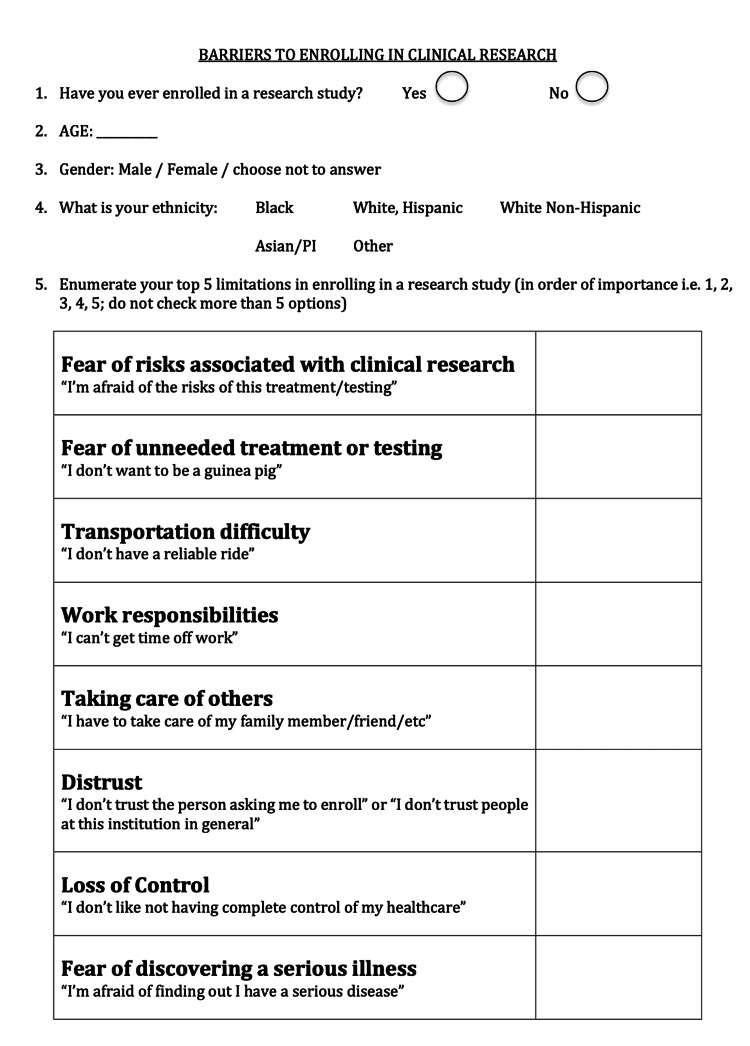
Study questionnaire.
